# Using scores from the 4AT delirium detection tool as an indicator of possible dementia: a study of 75 221 older adult hospital admissions

**DOI:** 10.1093/ageing/afaf144

**Published:** 2025-06-16

**Authors:** Rose S Penfold, Emily Bowman, Emma R L C Vardy, Elizabeth L Sampson, Atul Anand, Bruce Guthrie, Alasdair M J MacLullich

**Affiliations:** Usher Institute of Population Health Sciences and Informatics, The University of Edinburgh, NINE 9 Little France Road Edinburgh BioQuarter City, Edinburgh EH16 4UX, UK; Department of Psychiatry, University of Oxford, Oxford, UK; Royal Oldham Hospital, Northern Care Alliance NHS Foundation Trust, Rochdale Road, Oldham, OL1 2JH & Manchester Academic Health Sciences Centre, University of Manchester, Oxford Road, Manchester, M13 9PL, UK; Academic Centre for Healthy Ageing—Barts Health NHS Trust, Queen Mary University of London, London, UK; Centre for Psychiatry and Mental Health, Wolfson Institute of Population Health, Queen Mary University of London, London, UK; Centre for Cardiovascular Science, The University of Edinburgh College of Medicine and Veterinary Medicine, Rooms SU.305, Chancellor's Building 49 Little France Crescent, Edinburgh EH16 4TJ, UK; Advanced Care Research Centre, Usher Institute, University of Edinburgh, Edinburgh, UK; Ageing and Health, The University of Edinburgh Usher Institute of Population Health Sciences and Informatics, Edinburgh, UK

**Keywords:** dementia, delirium, acute care, hospital, assessment, older people

## Abstract

**Introduction:**

Overall dementia diagnosis rates are substantially below true rates. Hospital admissions of older people involve cognitive and functional assessments relevant to dementia diagnosis. These assessments could be harnessed to contribute to identifying patients for further assessment. Yet relationships of inpatient cognitive tests with known dementia are unclear. The 4AT (www.the4AT.com) assesses for delirium (Scores 4–12) and also cognitive impairment via embedded cognitive tests (Scores 1–3). We investigated relationships between 4AT scores and clinical dementia diagnoses.

**Methods:**

We included participants aged ≥65 years admitted as a medical emergency to three hospitals from 1 April 2016 to 1 April 2020, who had the 4AT performed on admission. Clinical dementia diagnosis was ascertained from linked primary care, hospital discharge and community prescribing data.

**Results:**

Of 75 221 admissions, 62 188 (82.7%; 33 625 unique patients; mean age 80.2 years; 55.8% female) had a 4AT on admission. Of these, 9948 (16.0%) had a recorded clinical dementia diagnosis at the time of admission, with a further 1197 (1.9%) receiving a new diagnosis at discharge. Of admissions with dementia, 9669/11 145 (86.8%) had a 4AT score ≥1 on admission, compared to 14 994/51 043 (29.4%) without dementia.

4AT ≥1 had a sensitivity of 0.87 (95% CI 0.86–0.87) and a specificity of 0.71 (0.70–0.71) in relation to clinical dementia diagnosis. 4AT ≥4 showed sensitivity of 0.50 (0.50–0.51) and a specificity of 0.88 (0.88–0.88).

**Conclusions:**

4AT scores were associated with clinically diagnosed dementia. These results suggest that routinely collected 4AT scores could be leveraged in conjunction with other clinical indicators to identify patients with possible undiagnosed dementia who could undergo further inpatient diagnostic assessment and/or post-discharge specialist follow-up.

## Key Points

- The 4AT assesses for delirium (Scores 4–12) and cognitive impairment via embedded cognitive tests (Scores 1–3).
- This study investigated the association between 4AT scores and clinically diagnosed dementia.
- Positive 4AT scores were associated with clinically diagnosed dementia.
- Routine 4AT scores could be used along with other clinical indicators to identify patients with possible undiagnosed dementia.

## Introduction

People with dementia are frequently hospitalised, with rates ranging from 0.37 to 1.26 per person-year in high-quality studies [1]. Dementia is present in at least 20% of older general hospital inpatients, yet over one-third lack a formal diagnosis [2–7]. Hospital admission of older people currently involves multiple cognitive and functional assessments, and these could be informative for dementia diagnosis. Admission presents a valuable potential opportunity to diagnose dementia and/or initiate a postdischarge diagnostic pathway.

National guidelines, including the 2012–2019 English Department of Health CQUIN framework, the Irish pathway for Suspected Dementia on Acute Hospital Wards and the 2023 Scottish Intercollegiate Guidelines Network (SIGN) dementia guideline, recommend dementia diagnosis in hospital for appropriate cases [8–10]. Existing studies support the feasibility of hospital-based diagnostic pathways. For example, one liaison psychiatry service in London received 2153 referrals for a range of psychiatric presentations over a 3-year period, of which 51 people (2%) were diagnosed with dementia in hospital and 273 (13%) referred for outpatient assessment [11]. However, hospital-based diagnosis remains relatively uncommon, for reasons including concerns around the interpretability of cognitive testing in hospital inpatients; inconsistent availability of specialists; and challenges in accessing postdiagnostic support, dementia drug prescription and monitoring pathways [12, 13].

Most hospital admissions occur for reasons other than dementia, and identifying possible dementia therefore relies on routine clinical assessments performed for other clinical purposes but which are potentially indicative of dementia. The 4 ‘A’s Test (4AT; www.the4AT.com) is widely used in UK hospitals and internationally for delirium detection. It is recommended for all older adult hospital admissions by National Health Service (NHS) England and by Scottish Intercollegiate Guidelines Network (SIGN) delirium guidelines [14, 15]. The 4AT is scored from 0 to 12. A 4AT score ≥4 is sensitive and specific for delirium, as demonstrated in multiple validation studies [16]. Delirium is a strong indicator of dementia, with dementia present in over half of inpatients with delirium [2, 6, 17]. However, unlike other delirium detection tools, as well as assessing for delirium, the 4AT also incorporates a ‘possible cognitive impairment’ category (Score 1–3). This is scored through two short embedded cognitive tests, the Abbreviated Mental Test 4 (AMT-4) and Months of the Year (MOTY) Backwards test, which are commonly used as simple tests of cognitive impairment [18, 19]. Thus, any 4AT score ≥1 could indicate possible dementia and prompt more detailed evaluation, when interpreted alongside other relevant clinical information.

Previous studies have demonstrated associations between 4AT scores of 1–3 and adverse outcomes including higher mortality, longer hospital stays and reduced time at home after discharge, but few have directly examined associations with dementia [20, 21]. A study of 419 older adults attending a single Emergency Department (ED) in Ireland found that a 4AT score ≥2 had a sensitivity of 0.74 (0.64–0.83) and a specificity of 0.87 (0.82–0.90) for dementia compared to expert clinical assessment [22]. In that study, the 4AT was administered by trained researchers. Whether the 4AT used in routine care can help to indicate dementia remains underexplored.

This study examined admission 4AT scores in relation to recorded dementia diagnoses, aiming to assess performance of the 4AT for identifying dementia, including dementia newly diagnosed during hospital admission. We hypothesised that a 4AT score ≥1 would demonstrate good sensitivity and specificity for clinically diagnosed dementia and thus could be used alongside other relevant clinical information to identify patients requiring further cognitive assessment.

## Methods

This retrospective cohort study used routinely collected pseudonymised data accessed via the DataLoch service (dataloch.org). We included people aged ≥65 with an emergency medical admission to three NHS Lothian hospitals from 4 January 2016 to 4 January 2020 with 4AT recorded on admission. All included participants were registered with a General Practitioner (GP) contributing to DataLoch (~90% of GP practices and ~86% of older adults in Lothian), with at least 1 year of GP registration before admission [23, 24].

### Data sources

Emergency hospitalisation data were obtained from the hospital EHR (TrakCare, Intersystems). Dementia diagnoses were determined using linked GP (Read version2 codes), hospital discharge and community prescribing records. Hospital discharge data were obtained through linkage to Scottish Morbidity Records 01 (SMR01), a national administrative dataset capturing all acute inpatient episodes, with up to six diagnoses recorded per episode using International Classification of Diseases 10th revision (ICD-10) codes. Dementia was identified in SMR01 and GP data using code lists from the HDR UK phenotype library (Appendix 1) [25]. Community prescription data were obtained from the Prescribing Information System, capturing all NHS Scotland community-dispensed prescriptions. Donepezil, galantamine, rivastigmine or memantine prescriptions were considered diagnostic for dementia, as these are only recommended for NHS prescription for dementia treatment by the Scottish Medicines Consortium.

The 4AT was performed on admission as part of routine care. Scores were categorised as per instructions (www.the4AT.com): 4AT 0 (no delirium), 4AT 1–3 (cognitive impairment without delirium) and 4AT ≥4 (possible delirium).

### Analyses

All analyses were performed using de-identified data in the Lothian DataLoch secure data environment, using R version 4.4.1.

All categorical variables are presented as frequencies and percentages and continuous variables as mean (standard deviation) or median (interquartile range). Performance of the 4AT was determined in relation to a pragmatic clinical reference standard of recorded formal (specialist) clinical dementia diagnoses in any data source, including new diagnoses recorded during hospital admission.

### Sensitivity analysis

We conducted a sensitivity analysis using only one admission per individual (randomly selected) to assess whether recurrent admissions influenced study findings.

### Ethics

The project received approval through DataLoch, including: Caldicott Guardian, ACCORD sponsorship (AC23107), favourable ethical opinion under DataLoch’s delegated authority (Reference: 23/NS/0093) and a public value assessment by the DataLoch Public Reference Group.

## Results

Of 75 221 admissions, 62 188 (82.7%; 33 625 unique patients; mean age 80.2 (SD 8.1) years; 55.8% female) had a 4AT score on admission (Table 1). Of these, 11 145 (17.9%) had a dementia diagnosis: 9948 (16.0%) recorded at the time of admission and 1197 (1.9%) newly recorded at discharge.

**Table 1:** Characteristics and 4AT scores of all admissions during the study period with a recorded 4AT score, stratified by dementia status

	All admissions^*^ *N* = 62 188	No dementia recorded *N* = 51 043	Dementia recorded on admission *N* = 9948	Dementia newly recorded at discharge *N* = 1197
**Age in years**				
Mean (SD)	80.2 (8.1)	79.3 (8.1)	84.1 (6.8)	85.0 (7.2)
**Sex** F (%)	34 708 (55.8)	28 048 (54.9)	5959 (59.9)	701 (58.6)
**SIMD quintile** ** N (%)				
1 (most deprived)	10 588 (17.1)	8790 (17.3)	1617 (16.3)	181 (15.1)
2	15 530 (25.1)	13 029 (25.6)	2231 (22.5)	270 (22.6)
3	10 395 (16.8)	8697 (17.1)	1528 (15.4)	170 (14.2)
4	10 224 (16.5)	8080 (15.9)	1883 (19.0)	261 (21.8)
5 (least deprived) *231 missing*	15 220 (24.6)	12,251 (24.1)	2656 (26.8)	313 (26.2)
**Ethnicity** *N* (%)				
White	57 847 (96.0)	47 411 (95.9)	9341 (96.5)	1095 (95.8)
Other ethnic group *1932 missing*	2409 (4.0)	2021 (4.1)	340 (3.5)	48 (4.2)
**4AT score on admission** *N* (%)				
0	37 525 (60.3)	36 049 (70.6)	1327 (13.3)	149 (12.5)
1–3	12 912 (20.8)	8803 (17.2)	3630 (36.5)	479 (40.0)
4+	11 751 (18.9)	6191 (12.1)	4991 (50.2)	569 (47.5)
**4AT components** *N* (%) **Alertness**				
Normal (0)	55 888 (89.9)	47 479 (93.0)	7483 (75.2)	935 (78.1)
Mild sleepiness <10 s, then normal (0)	3158 (5.1)	1995 (3.9)	1051 (10.6)	112 (9.4)
Clearly abnormal (4)	3142 (5.1)	1578 (3.1)	1414 (14.2)	150 (12.5)
**AMT4**				
No mistakes (0)	42 403 (68.2)	40 107 (78.6)	2044 (20.5)	252 (21.1)
1 mistake (1)	7268 (11.7)	5061 (9.9)	1914 (19.2)	293 (24.5)
2 or more mistakes/ untestable (2)	12 517 (20.1)	5875 (11.5)	5990 (60.2)	652 (54.5)
**Attention**				
7 MOTY backwards correctly (0)	40 926 (65.8)	38 625 (75.7)	2055 (20.7)	246 (20.6)
Starts but <7 MOTY backwards correct (1)	12 562 (20.2)	8161 (16.0)	3825 (38.4)	576 (48.1)
Untestable (2)	8700 (14.0)	4257 (8.3)	4068 (40.9)	375 (31.3)
**Acute change or fluctuating course**				
No (0)	54 823 (88.2)	46 819 (91.7)	7177 (72.1)	827 (69.1)
Yes (4)	7365 (11.8)	4224 (8.3)	2771 (27.9)	370 (30.9)

^a^Of 75 221 admissions during the study period, 13 033 (17.3%) had no recorded 4AT score: 1659 (14.3%) with dementia recorded on admission, 11 278 (18.1%) with no dementia and 96 (7.4%) with dementia newly recorded at discharge.

^b^SIMD, Scottish Index of Multiple Deprivation, an area-based measure of relative social deprivation.

Of the 11 145 admissions with dementia, 9669 (86.7%) had a 4AT score ≥1 on admission, compared to 14 994/51 043 (29.4%) of those without recorded dementia (Table 1). The distribution of 4AT scores was similar in those with dementia recorded at admission and those newly diagnosed in hospital. Of admissions with dementia, 8849 (79.4%) and 8844 (79.4%) had abnormal scores on the AMT4 and MOTY Backwards components of the 4AT, respectively, compared to 10 936 (21.4%) and 12 418 (24.3%) of admissions without dementia.

The estimated diagnostic accuracy of the 4AT in relation to a recorded clinical dementia diagnosis is shown in Table 2 at different score thresholds. A 4AT ≥1 demonstrated a sensitivity of 0.87 (0.86–0.87) and a specificity of 0.71 (0.70–0.71). Increasing the 4AT score cutoff increased specificity but reduced sensitivity. At the ‘possible delirium’ cutoff of ≥4, approximately half of those with dementia would be missed.

**Table 2:** Diagnostic test accuracy of the 4AT relative to the reference standard of a clinical dementia diagnosis recorded in any data source

	Number screening positive at threshold (% of all admissions)	Sensitivity (95% CI)	PPV (95% CI)	Specificity (95% CI)	NPV (95% CI)
**4AT** ≥**1**	24 663 (39.7)	0.87 (0.86–0.87)	0.39 (0.39–0.40)	0.71 (0.70–0.71)	0.96 (0.96–0.96)
**4AT** ≥**2**	19 074 (30.7)	0.78 (0.77–0.78)	0.45 (0.45–0.46)	0.80 (0.79–0.80)	0.94 (0.94–0.94)
**4AT** ≥**3**	14 865 (23.9)	0.65 (0.64–0.66)	0.49 (0.48–0.49)	0.85 (0.85–0.85)	0.92 (0.91–0.92)
**4AT** ≥**4**	11 751 (18.9)	0.50 (0.50–0.51)	0.47 (0.46–0.48)	0.88 (0.88–0.88)	0.89 (0.89–0.89)

PPV, positive predictive value; NPV, negative predictive value

### Sensitivity analysis

Findings of an individual-based sensitivity analysis were consistent with the primary admissions–based analysis (Appendices 2 and 3).

## Discussion

In this large population cohort routine data study, we found that 4AT scores in both the cognitive impairment (1–3) and delirium (≥4) ranges were associated with clinically recorded dementia. A 4AT score ≥1 demonstrated good sensitivity and acceptable specificity for identifying dementia, with increasing specificity at higher cutoffs, including in the delirium range. Our findings align with those reported in a study of 419 over-70s attending a single ED, where trained researchers performed the 4AT and specialists evaluated all study participants for dementia [22].

Nearly 40% of study participants had a 4AT ≥1, and around 40% of these had a dementia diagnosis. Higher scoring thresholds demonstrated higher specificity for clinical dementia but lower sensitivity. We do not propose that the 4AT is diagnostic for dementia or is a standalone dementia screening tool. Instead, we suggest that a 4AT score ≥1 should be interpreted as one useful potential indicator of dementia, alongside other relevant information including informant history, clinical context and other assessments performed during admission. Dementia diagnosis remains the role of specialists, but the 4AT, already widely implemented for delirium detection, could help identify patients for further in-hospital assessment or referral. [14, 15, 26, 27].

Study strengths include the large, consecutive cohort of unselected, unscheduled medical admissions. 4AT assessments were performed in routine care across three hospitals, increasing generalisability. Clinically recorded dementia diagnoses were likely accurate, with a previous study in the same geographical region reporting a positive predictive value of 87.3% for recorded dementia diagnoses compared to expert adjudication of medical records [28]. One limitation is the use of recorded dementia diagnoses as the pragmatic reference standard, because undiagnosed individuals are included in the no dementia comparison group. However, we have shown that in this population, combining primary care, hospital discharge and community-prescribing records captures ~80% of estimated dementia cases [29]. Overall, the undiagnosed patients are therefore a small proportion of the no dementia group used in analyses in this study. We were unable to test 4AT scores in relation to both diagnosed and undiagnosed dementia in all eligible patients, unlike in the study by O’Sullivan *et al*. [22]. HDR UK dementia phenotype codes do not include dementia with Lewy bodies, although this comprises ~5% of recorded dementia [25, 30]. Another limitation is that 17% of patients did not have a 4AT performed. However, a large majority of patients in the study had both 4AT and clinical dementia ascertainment, and, overall, the pattern of results is similar to that reported in the prior prospective study [22]. We used an admissions-based approach, which may overrepresent individuals with multiple admissions who are more likely to have dementia. However, this approach reflects real-world diagnostic practices, and an individual-based sensitivity analysis showed similar findings.

Future work could explore combining 4AT scores with other readily available variables such as frailty scores, age thresholds and comorbidities to identify patients who may benefit from more detailed assessment. Use of such a collection of indicators could be deployed in different ways depending on local context, availability of specialists and resources in the hospital and the community. The current analysis could also be extended to explore the association between specific domains of the 4AT (notably AMT4 and MOTY backwards) and dementia diagnoses.

## Conclusion

The 4AT is easy to implement and already widely used globally for delirium detection. Our findings suggest 4AT scores could also be leveraged alongside other clinical indicators to identify hospitalised patients with possible dementia requiring further inpatient specialist diagnostic assessment and/or post-discharge follow-up.

## Supplementary Material

aa-24-2760-File002_afaf144

## Data Availability

Data may be accessed through DataLoch (dataloch.org) following successful application and approvals.

## References

[1] Shepherd H, Livingston G, Chan J et al. Hospitalisation rates and predictors in people with dementia: A systematic review and meta-analysis. BMC Med 2019;17:130. 10.1186/s12916-019-1369-7. 31303173 PMC6628507

[2] Timmons S, Manning E, Barrett A et al. Dementia in older people admitted to hospital: A regional multi-hospital observational study of prevalence, associations and case recognition. Age Ageing 2015;44:993–9. 10.1093/ageing/afv131. 26420638 PMC4621233

[3] Bickel H, Hendlmeier I, Heßler JB et al. The prevalence of dementia and cognitive impairment in hospitals. Dtsch Arztebl Int 2018;115:733–40. 10.3238/arztebl.2018.0733. 30565543 PMC6318438

[4] Mukadam N, Sampson EL. A systematic review of the prevalence, associations and outcomes of dementia in older general hospital inpatients. Int Psychogeriatr 2011;23:344–55. 10.1017/S1041610210001717. 20716393

[5] Briggs R, Dyer A, Nabeel S et al. Dementia in the acute hospital: The prevalence and clinical outcomes of acutely unwell patients with dementia. QJM 2017;110:33–7. 10.1093/qjmed/hcw114. 27486262

[6] Jackson TA, MacLullich AM, Gladman JR et al. Undiagnosed long-term cognitive impairment in acutely hospitalised older medical patients with delirium: A prospective cohort study. Age Ageing 2016;45:493–9. 10.1093/ageing/afw064. 27076525

[7] Sampson EL, Blanchard MR, Jones L et al. Dementia in the acute hospital: Prospective cohort study of prevalence and mortality. Br J Psychiatry 2009;195:61–6. 10.1192/bjp.bp.108.055335. 19567898

[8] NHS England . Commissioning for Quality and Innovation (CQUIN): 2014/15 Guidance. 2014; https://www.england.nhs.uk/wp-content/uploads/2014/12/sc-cquin-guid.pdf.

[9] Health Service Executive (2020). Diagnostic Pathway for Suspected Dementia on Acute Hospital Wards. https://www.hse.ie/eng/dementiapathways/files/diagnostic-pathway-for-suspected-dementia-on-acute-hospitalwards.pdf [13 October 2024, date last accessed].

[10] Healthcare Improvement Scotland . 2023. Assessment, diagnosis, care and support for people with dementia and their carers. https://www.sign.ac.uk/our-guidelines/dementia/ 30 January 2024, date last accessed.

[11] Mujic F, Cairns R, Mak V et al. Liaison psychiatry for older adults in the general hospital: Service activity, development and outcomes. BJPsych Bull 2018;42:30–6. 10.1192/bjb.2017.9. 29388526 PMC6001875

[12] Russ TC, Shenkin SD, Reynish E et al. Dementia in acute hospital inpatients: The role of the geriatrician. Age Ageing 2012;41:282–4. 10.1093/ageing/afs048. 22454135

[13] Xiong B, Bailey DX, Prudon P et al. Identification and information management of cognitive impairment of patients in acute care hospitals: An integrative review. Int J Nurs Sci 2024;11:120–32. 10.1016/j.ijnss.2023.11.001. 38352291 PMC10859579

[14] Scottish Intercollegiate Guidelines Network (SIGN) . Risk reduction and management of delirium. Edinburgh: SIGN; 2019. (SIGN publication no. 157). 2019. http://www.sign.ac.uk. 10.3390/medicina55080491 PMC6722546 31443314

[15] Hopper A . 2021. Geriatric Medicine: Getting It Right First Time National Specialty Report. https://gettingitrightfirsttime.co.uk/wp-content/uploads/2021/09/Geriatric-Medicine-Sept21h.pdf 31 October 2024, date last accessed.

[16] Tieges Z, Maclullich AMJ, Anand A et al. Diagnostic accuracy of the 4AT for delirium detection in older adults: Systematic review and meta-analysis. Age Ageing 2021;50:733–43. 10.1093/ageing/afaa224. 33951145 PMC8099016

[17] Pendlebury ST, Lovett NG, Smith SC et al. Observational, longitudinal study of delirium in consecutive unselected acute medical admissions: Age-specific rates and associated factors, mortality and re-admission. BMJ Open 2015;5:e007808. 10.1136/bmjopen-2015-007808. PMC4654280 26576806

[18] Carpenter CR, Banerjee J, Keyes D et al. Accuracy of dementia screening instruments in emergency medicine: A diagnostic meta-analysis. Acad Emerg Med 2019;26:226–45. 10.1111/acem.13573. 30222232

[19] Hasemann W, Duncan N, Clarke C et al. Comparing performance on the months of the year backwards test in hospitalised patients with delirium, dementia, and no cognitive impairment: An exploratory study. Eur Geriatr Med 2021;12:1257–65. 10.1007/s41999-021-00521-4. 34156656 PMC8626373

[20] Anand A, Cheng M, Ibitoye T et al. Positive scores on the 4AT delirium assessment tool at hospital admission are linked to mortality, length of stay and home time: Two-Centre study of 82,770 emergency admissions. Age Ageing 2022;51. 10.1093/ageing/afac051. PMC8923813 35292792

[21] Penfold RS, Hall AJ, Anand A et al. Delirium in hip fracture patients admitted from home during the COVID-19 pandemic is associated with higher mortality, longer total length of stay, need for post-acute inpatient rehabilitation, and readmission to acute services. Bone Jt Open 2023;4:447–56. 10.1302/2633-1462.46.BJO-2023-0045.R1. 37326476 PMC10274512

[22] O'Sullivan D, Brady N, Manning E et al. Validation of the 6-item cognitive impairment test and the 4AT test for combined delirium and dementia screening in older emergency department attendees. Age Ageing 2018;47:61–8. 10.1093/ageing/afx149. 28985260 PMC5860384

[23] NRS . Scotland's Census 2022 - Rounded population estimates. 2022 [updated September 14th, 2023.; cited 2024 June 19th]; https://www.scotlandscensus.gov.uk/2022-results/scotland-s-census-2022-rounded-population-estimates/.

[24] Lewis JD, Bilker WB, Weinstein RB et al. The relationship between time since registration and measured incidence rates in the general practice research database. Pharmacoepidemiol Drug Saf 2005;14:443–51. 15898131 10.1002/pds.1115

[25] Kuan V, Denaxas S, Gonzalez-Izquierdo A et al. A chronological map of 308 physical and mental health conditions from 4 million individuals in the English national health service. Lancet Digit Health 2019;1:e63–77. 10.1016/S2589-7500(19)30012-3. 31650125 PMC6798263

[26] Tieges Z, Lowrey J, MacLullich AMJ. What delirium detection tools are used in routine clinical practice in the United Kingdom? Survey results from 91% of acute healthcare organisations. Eur Geriatr Med 2021;12:1293–8. 10.1007/s41999-021-00507-2. 34008099 PMC8626368

[27] Penfold RS, Squires C, Angus A et al. Delirium detection tools show varying completion rates and positive score rates when used at scale in routine practice in general hospital settings: A systematic review. J Am Geriatr Soc 2024;72:1508–24. 10.1111/jgs.18751. 38241503

[28] Wilkinson T, Schnier C, Bush K et al. Identifying dementia outcomes in UK biobank: A validation study of primary care, hospital admissions and mortality data. Eur J Epidemiol 2019;34:557–65. 10.1007/s10654-019-00499-1. 30806901 PMC6497624

[29] Penfold RS, Wilkinson T, Russ T, Stirland L, MacRae C, Shenkin S., et al. Prevalence and Outcomes of Recorded Dementia: a Population-Based Study of 133,407 Older Adults using Linked Routine Data Sources. https://www.bgs.org.uk/sites/default/files/content/attachment/2024-12-02/2024_bgs_autumn_abstract_book.pdf. 10.1093/ageing/afag038 41734099

[30] Kane JPM, Surendranathan A, Bentley A et al. Clinical prevalence of Lewy body dementia. Alzheimers Res Ther 2018;10:119. 10.1186/s13195-018-0350-6. 29448953 PMC5815202

