# Correction to: Using scores from the 4AT delirium detection tool as an indicator of possible dementia: a study of 75 221 older adult hospital admissions

**DOI:** 10.1093/ageing/afag134

**Published:** 2026-05-04

This is a correction to: Rose S Penfold, Emily Bowman, Emma R L C Vardy, Elizabeth L Sampson, Atul Anand, Bruce Guthrie, Alasdair M J MacLullich, Using scores from the 4AT delirium detection tool as an indicator of possible dementia: a study of 75 221 older adult hospital admissions, *Age and Ageing*, Volume 54, Issue 6, June 2025, afaf144, https://doi.org/10.1093/ageing/afaf144

In the originally published version of this manuscript, the study dates were erroneously listed as “4 January 2016 to 4 January 2020” in the Abstract and the ‘Methods’ section.

These dates have been corrected to read “1 April 2016 to 1 April 2020”.

