# Supplementary material for: Using scores from the 4AT delirium detection tool as an indicator of possible dementia: a study of 75 221 older adult hospital admissions
Source: Age Ageing. 2025 Jun 16;54(6):afaf144. doi: 10.1093/ageing/afaf144 (PMC12167849; doi:10.1093/ageing/afaf144)
Supplement: aa-24-2760-File002_afaf144 [file aa-24-2760-file002_afaf144.docx]

**Supplementary Materials**

**Using Scores from the 4AT Delirium Detection Tool as an Indicator of Possible Dementia: A Study of 75,221 Older Adult Hospital Admissions**

Appendix 1**.** Read version 2 and ICD-10 code lists for dementia.

Source: HDR UK Phenotype library, available at <https://phenotypes.healthdatagateway.org/phenotypes/PH148/version/296/detail/>

| **code** | **description** | **coding_system** |
| --- | --- | --- |
| **Eu01000** | [X]Vascular dementia of acute onset | Read codes v2 |
| **1461.00** | H/O: dementia | Read codes v2 |
| **E00y.11** | Presbyophrenic psychosis | Read codes v2 |
| **F110.00** | Alzheimer's disease | Read codes v2 |
| **E001.00** | Presenile dementia | Read codes v2 |
| **F110100** | Alzheimer's disease with late onset | Read codes v2 |
| **Eu01y00** | [X]Other vascular dementia | Read codes v2 |
| **Eu00000** | [X]Dementia in Alzheimer's disease with early onset | Read codes v2 |
| **E00y.00** | Other senile and presenile organic psychoses | Read codes v2 |
| **E00..00** | Senile and presenile organic psychotic conditions | Read codes v2 |
| **E001z00** | Presenile dementia NOS | Read codes v2 |
| **9Ou2.00** | Dementia monitoring second letter | Read codes v2 |
| **9hD0.00** | Excepted from dementia quality indicators: Patient unsuitabl | Read codes v2 |
| **Eu01111** | [X]Predominantly cortical dementia | Read codes v2 |
| **Eu00200** | [X]Dementia in Alzheimer's dis, atypical or mixed type | Read codes v2 |
| **Eu00z11** | [X]Alzheimer's dementia unspec | Read codes v2 |
| **E003.00** | Senile dementia with delirium | Read codes v2 |
| **E041.00** | Dementia in conditions EC | Read codes v2 |
| **E004.00** | Arteriosclerotic dementia | Read codes v2 |
| **E004100** | Arteriosclerotic dementia with delirium | Read codes v2 |
| **E002100** | Senile dementia with depression | Read codes v2 |
| **Eu01.11** | [X]Arteriosclerotic dementia | Read codes v2 |
| **E002z00** | Senile dementia with depressive or paranoid features NOS | Read codes v2 |
| **66h..00** | Dementia monitoring | Read codes v2 |
| **Eu02z11** | [X] Presenile dementia NOS | Read codes v2 |
| **Eu01.00** | [X]Vascular dementia | Read codes v2 |
| **E002.00** | Senile dementia with depressive or paranoid features | Read codes v2 |
| **8CMZ.00** | Dementia care plan | Read codes v2 |
| **Eu02z14** | [X] Senile dementia NOS | Read codes v2 |
| **E001200** | Presenile dementia with paranoia | Read codes v2 |
| **Eu01300** | [X]Mixed cortical and subcortical vascular dementia | Read codes v2 |
| **Eu01z00** | [X]Vascular dementia, unspecified | Read codes v2 |
| **Eu02z15** | [X] Senile psychosis NOS | Read codes v2 |
| **E004200** | Arteriosclerotic dementia with paranoia | Read codes v2 |
| **E004300** | Arteriosclerotic dementia with depression | Read codes v2 |
| **6AB..00** | Dementia annual review | Read codes v2 |
| **Eu00011** | [X]Presenile dementia,Alzheimer's type | Read codes v2 |
| **Eu00111** | [X]Alzheimer's disease type 1 | Read codes v2 |
| **E004.11** | Multi infarct dementia | Read codes v2 |
| **Eu02z12** | [X] Presenile psychosis NOS | Read codes v2 |
| **Eu00012** | [X]Primary degen dementia, Alzheimer's type, presenile onset | Read codes v2 |
| **Eu04100** | [X]Delirium superimposed on dementia | Read codes v2 |
| **E00..12** | Senile/presenile dementia | Read codes v2 |
| **E004z00** | Arteriosclerotic dementia NOS | Read codes v2 |
| **Eu02z13** | [X] Primary degenerative dementia NOS | Read codes v2 |
| **F110000** | Alzheimer's disease with early onset | Read codes v2 |
| **E002000** | Senile dementia with paranoia | Read codes v2 |
| **Eu00.00** | [X]Dementia in Alzheimer's disease | Read codes v2 |
| **Eu00113** | [X]Primary degen dementia of Alzheimer's type, senile onset | Read codes v2 |
| **Eu00z00** | [X]Dementia in Alzheimer's disease, unspecified | Read codes v2 |
| **9Ou4.00** | Dementia monitoring verbal invite | Read codes v2 |
| **9hD1.00** | Excepted from dementia quality indicators: Informed dissent | Read codes v2 |
| **9Ou5.00** | Dementia monitoring telephone invite | Read codes v2 |
| **9Ou3.00** | Dementia monitoring third letter | Read codes v2 |
| **9Ou1.00** | Dementia monitoring first letter | Read codes v2 |
| **Eu02z00** | [X] Unspecified dementia | Read codes v2 |
| **Eu00112** | [X]Senile dementia,Alzheimer's type | Read codes v2 |
| **Eu01100** | [X]Multi-infarct dementia | Read codes v2 |
| **9hD..00** | Exception reporting: dementia quality indicators | Read codes v2 |
| **E001000** | Uncomplicated presenile dementia | Read codes v2 |
| **E001300** | Presenile dementia with depression | Read codes v2 |
| **Eu01200** | [X]Subcortical vascular dementia | Read codes v2 |
| **ZS7C500** | Language disorder of dementia | Read codes v2 |
| **E00z.00** | Senile or presenile psychoses NOS | Read codes v2 |
| **E00..11** | Senile dementia | Read codes v2 |
| **Fyu3000** | [X]Other Alzheimer's disease | Read codes v2 |
| **Eu00100** | [X]Dementia in Alzheimer's disease with late onset | Read codes v2 |
| **E001100** | Presenile dementia with delirium | Read codes v2 |
| **E004000** | Uncomplicated arteriosclerotic dementia | Read codes v2 |
| **9Ou..00** | Dementia monitoring administration | Read codes v2 |
| **Eu02z16** | [X] Senile dementia, depressed or paranoid type | Read codes v2 |
| **Eu00013** | [X]Alzheimer's disease type 2 | Read codes v2 |
| **E000.00** | Uncomplicated senile dementia | Read codes v2 |
| **F05.1** | Delirium superimposed on dementia | ICD10 codes |
| **G30** | Alzheimer's disease | ICD10 codes |
| **F03** | Unspecified dementia | ICD10 codes |
| **F01** | Vascular dementia | ICD10 codes |
| **F00** | Dementia in Alzheimer's disease | ICD10 codes |

Appendix 2. Characteristics and 4AT scores of unique individuals during the study period (random admission) with a recorded 4AT score, stratified by dementia status.

|  | **All***  N=31381 | **No dementia recorded**  N=25972 | **Dementia recorded on admission**  N=4729 | **Dementia newly recorded at discharge**  N=680 |
| --- | --- | --- | --- | --- |
| **Age in years**  mean (SD) | 80.0 (8.2) | 79.0 (8.2) | 84.4 (6.8) | 85.2 (7.4) |
| **Sex** F(%) | 17432 (55.5) | 14144 (54.5) | 2873 (60.8) | 415 (61.0) |
| **SIMD quintile**** N(%)  1 (most deprived)  2  3  4  5 (least deprived)  *110 missing* | 4807 (15.4)  7616 (24.4)  5176 (16.6)  5292 (16.9)  8380 (26.8) | 4030 (15.6)  6437 (24.9)  4366 (16.9)  4196 (16.2)  6847 (26.5) | 669 (14.2)  1037 (22.0)  715 (15.2)  942 (20.0)  1353 (28.7) | 108 (15.9)  142 (20.9)  95 (14.0)  154 (22.7)  180 (26.5) |
| **Ethnicity** N(%)  White  Other ethnic group  *1308 missing* | 28723 (95.5)  1350 (4.5) | 23726 (95.4)  1138 (4.6) | 4387 (96.0)  182 (4.0) | 610 (95.3)  30 (4.7) |
| **4AT score on admission** N(%)  0  1-3  4+ | 19379 (61.8)  5884 (18.8)  6118 (19.5) | 18802 (72.4)  4023 (15.5)  3147 (12.1) | 494 (10.4)  1591 (33.6)  2644 (55.9) | 83 (12.2)  270 (39.7)  327 (48.1) |
| **4AT components** N(%)  **Alertness**  Normal (0)  Mild sleepiness <10 secs, then normal (0)  Clearly abnormal (4)  **AMT4**  No mistakes (0)  1 mistake (1)  2 or more mistakes/ untestable (2)  **Attention**  7 MOTY backwards correctly (0)  Starts but <7 MOTY backwards correct (1)  Untestable (2)  **Acute change or fluctuating course**  No (0)  Yes (4) | 28045 (89.4)  1554 (5.0)  1782 (5.7)  21589 (68.8)  3298 (10.5)  6494 (20.7)  20973 (66.8)  5848 (18.6)  4560 (14.5)  27516 (87.7)  3865 (12.3) | 24147 (93.0)  970 (3.7)  855 (3.3)  20661 (79.6)  2327 (9.0)  2984 (11.5)  20011 (77.0)  3799 (14.6)  2162 (8.3)  23768 (91.5)  2204 (8.5) | 3385 (71.6)  516 (10.9)  828 (17.5)  792 (16.7)  817 (17.3)  3120 (66.0)  829 (17.5)  1729 (36.6)  2171 (45.9)  3272 (69.2)  1457 (30.8) | 513 (75.4)  68 (10.0)  99 (14.6)  136 (20.0)  154 (22.6)  390 (57.4)  133 (19.6)  320 (47.1)  227 (33.4)  476 (70.0)  204 (30.0) |

*Of 38,254 unique individuals admitted during the study period, on a randomly selected admission, 6873 (18.0%) had no recorded 4AT score recorded: 804 (19.0%) with dementia recorded on admission, 6071 (14.6%) with no dementia, and 49 (6.7%) with dementia newly recorded at discharge.

**SIMD: Scottish Index of Multiple Deprivation, an area-based measure of relative social deprivation.

Appendix 3. Diagnostic test accuracy of the 4AT relative to the reference standard of a recorded dementia diagnosis in any data source, including unique individuals admitted during the study period.

|  | **Number screening positive at threshold**  **(% of patients)** | **Sensitivity**  **(95% CI)** | **PPV**  **(95% CI)** | **Specificity**  **(95% CI)** | **NPV**  **(95% CI)** |
| --- | --- | --- | --- | --- | --- |
| **4AT** ≥ **1** | 12002 (38.2) | 0.89 (0.88-0.90) | 0.40 (0.39-0.41) | 0.72 (0.72-0.73) | 0.97 (0.97-0.97) |
| **4AT** ≥ **2** | 9511 (30.3) | 0.81 (0.80-0.82) | 0.46 (0.45-0.47) | 0.80 (0.80-0.81) | 0.95 (0.95-0.96) |
| **4AT** ≥ **3** | 7624 (24.3) | 0.70 (0.69-0.71) | 0.50 (0.48-0.51) | 0.85 (0.85-0.86) | 0.93 (0.93-0.93) |
| **4AT** ≥ **4** | 6118 (19.5) | 0.55 (0.54-0.56) | 0.49 (0.47-0.50) | 0.88 (0.87-0.88) | 0.90 (0.90-0.91) |

PPV: positive predictive value. NPV: negative predictive value
